# Relationship Between Emotional States, Emotion Regulation and Executive Functions in Professional Female Football Players

**DOI:** 10.3390/sports14070268

**Published:** 2026-06-28

**Authors:** Alan de Jesús Gómez-Rosales, Xóchitl Angélica Ortiz-Jiménez, Javier Sanchez-Lopez

**Affiliations:** 1 Universidad Autónoma de Nuevo León, Facultad de Psicología, San Nicolás de los Garza 66455, Mexico; alan.gomezrsl@uanl.edu.mx; 2 Escuela Nacional de Estudios Superiores Unidad Juriquilla, Universidad Nacional Autónoma de México, Santiago de Queretaro 76230, Mexico; javier.sanchezlopez@unam.mx

**Keywords:** football, anxiety, depression, cognitive flexibility, cognition

## Abstract

Football performance depends on multiple interacting factors, including physical, technical, tactical, and psychological components. Among the psychological factors associated with optimal performance are athletes’ emotional states, their regulation, and executive functions. Although executive functions and emotional states have been widely studied in sport settings, research examining the relationship between these variables in athletes is limited, particularly in female football players. The aim of this study was to explore the relationship between emotional states, emotional regulation, and performance on cognitive tasks in female players from the Mexican football league. Twenty-eight players participated in two individual assessment sessions in which anxiety and depression levels, emotional regulation, and executive functions—planning, inhibitory control, working memory, and cognitive flexibility—were evaluated using psychological and neuropsychological tests. Results indicated a positive correlation between decision-making and emotional attention (rho = 0.36; *p* < 0.05), as well as between depression levels and onset latency in a working memory task (rho = 0.38; *p* < 0.04). Finally, a negative correlation was identified between the percentage of risk cards and the TMMS attention score (rho = −0.47; *p* < 0.01). These findings suggest associations between emotional processes and cognitive functioning in professional female football players and warrant further investigation in sport-performance settings.

## 1. Introduction

To succeed in sports, not only physical abilities but also cognitive and emotional skills are required to enhance performance [1]. In fact, athletes must process and respond to large amounts of information within a short period of time and under high-pressure conditions. As a result, sports such as football emphasize the importance of these processes and states due to the social and cultural surroundings. Football demands players develop new skills and abilities depending on the position they play [2]. Several authors suggest that performance indicators on football can be categorized into three different factors: physical or physiological, psychological/contextual, and technical–tactical [3].

Among psychological skills, executive functions (EFs) play a central role. EFs are the abilities that control, regulate, and plan behavior and cognitive processes [4]. They help with focusing, paying attention, and thinking [5], regulating behavior and thoughts [6]. Miyake et al. [7] found that decision-making, inhibitory control, working memory, planning, and cognitive flexibility are core EFs. Years later, Vestberg et al. [8] classified EFs as higher-level (mental flexibility, inhibitory control, decision-making) and basic (e.g., working memory) categories. On the other hand, Zelazo & Müller [9] propose that executive functions can be categorized as ‘cold’ and ‘hot’. Cold EFs are specifically related to cognitive processes that do not depend on emotions, such as working memory, cognitive flexibility, and inhibitory control, while ‘hot’ EFs relate to emotional processes in interaction with specific contexts, primarily encompassing decision-making.

To be successful in football, a good functioning of hot and cold EFs is necessary because they interact with each other and with other psychological factors, like emotional state. Emotional regulation is crucial for adapting behavior to an individual’s context and goals, so its relationship with EF appears to be fundamental [10]. EFs have a role in regulating emotional responses to positive and negative emotions [11] as well as stressful stimuli [12]. Inhibitory control influences emotional expression [13] and working memory contributes to emotion regulation [14]. Furthermore, emotions such as frustration can negatively impact EF, leading to decreased performance in cognitive tasks [15].

Like cognitive processes, emotions play a fundamental role in athletic performance. Emotional regulation is necessary for optimal performance, particularly at high levels of competition, where cognitive, tactical, and technical aspects can be diminished or blocked by negative emotions or by lacking control over them [16]. Estrada Contreras & Córdoba [17] stated that higher levels of anxiety and stress tend to negatively impact EF capacity. In fact, football players who score higher on the anxiety scale may exhibit behaviors related to low self-control, including verbal and physical aggression [18].

Emotional reactivity and negative feelings affect cognitive performance [19], and depression is prevalent among individuals with cognitive impairment [20]. The development of the relationship between emotions and EF alongside biological maturation reinforces and influences both over time [21]. This makes their interaction essential for athletic performance. Accumulating evidence supports the importance of neurocognitive functioning for male and female football players’ success [8,22,23]. Therefore, developing these abilities becomes crucial for an optimal performance-oriented football approach. EFs such as emotional regulation interact with decision-making processes that optimize the physical, technical and tactical aspects of football [24], impacting performance significantly.

As noted earlier, rational decision-making involves emotional regulation, given its role in orienting toward available options [25]. Soylu [26] found that differences in emotional regulation abilities among football players may be influenced by the game’s demands. Players with greater emotional regulation tended to exhibit better adaptability in competitive settings. This highlights the importance of emotional regulation in athletic performance and its potential impact on cognitive functioning in sports contexts.

According to FIFA’s statistics, more than 30 million women currently practice football, a sport that is continuously growing among the female population [27]. In several countries, including Mexico, part of this growth has been driven by supporting the national women’s football league. However, few studies have examined emotional regulation and executive functions in female football players, and even fewer have focused on professional level.

Moreover, most studies examining EF in women’s football primarily focus on concussion development and cognitive changes after heading [28]. Additionally, some researchers have aimed to establish valid evaluation protocols for the female population, enabling the measurement of both simple and complex EF and their relation to players’ performance [23]. In the psychoaffective field, several studies suggest that training loads [29], exercise intensity [30], and match results [31] influence players’ emotional states, and constant monitoring can enhance sport performance [32]. Furthermore, professional female football players tend to have higher anxiety levels compared to amateur players [33], and their self-perceived performance influences their ability to regulate these states [34].

Given the extensive literature on the importance of EF in daily life and sports, and the crucial role of emotions in athletic performance, the interaction between these factors warrants further investigation. Although the relationship between emotions and EF has been well-documented in the general population, studies focusing on athletes are rare, especially among female football players. Therefore, research on this field is essential for understanding the cognitive and emotional development of female football players. The aim of this study was to explore the relationship between emotional states and performance in executive function tasks among professional female football players in the Mexican league. We hypothesize that there is a relationship between emotional states and performance on executive function tasks, and between emotional regulation and executive function scores. We also expect football female players to score above the mean on each neuropsychological test.

## 2. Materials and Methods

### 2.1. Study Design

In adherence to the Declaration of Helsinki, the research protocol was designed as a cross-sectional, exploratory and correlational study. This study was submitted to the ethics committee of the Faculty of Sports Organization at the Autonomous University of Nuevo León, with identification number REPRIN-FOD-167.

### 2.2. Participants

Twenty-eight professional female football players from the MX league participated, with a mean age (SD) of 22.18 (±4.8) years and a range of 16–30 years. The player’s average years of education was 13.61 years (this is important for normative neuropsychological test scores). Participants had played football in different positions, between 1 and 24 years with an average of 13.46 (±5.54). For this study, these positions were divided into goalkeepers (*n* = 4), defenders (*n* = 8), midfielders (*n* = 9), and attackers (*n* = 7). The inclusion criteria were informed consent signed by each participant and their parents in the case of minors, being registered with the club in the Mexican league and scoring above 26 in on the cognitive screening to rule out cognitive impairment using the Montreal Cognitive Assessment (MoCA; [35]). The exclusion criteria encompassed the presence of a documented psychiatric history and/or traumatic injuries. None of the 28 participants were excluded from the study.

### 2.3. Instruments

#### 2.3.1. Executive Functions Assessment

Iowa Gambling Task. This test evaluates decision-making, impulsivity, and risk-taking under uncertainty by assessing the risks and benefits of choices. Participants choose a card, earning points. However, they may lose points if the evaluator shows a card with penalties. The test provides two scores: the total score (sum of points minus penalties) and the percentage of risk cards (proportion of 4- and 5-point cards relative to total chosen; [36]). Higher scores indicate better performance.

Corsi’s Cube Test. Evaluates visuospatial processing and working memory. Participants point to marked cubes in reverse order. Scores analyzed include maximum retained items and onset latency (time to start each trial after the last cube lights up; [37]). Higher scores indicate better performance.

Wisconsin Card Sorting Test. It is designed to measure mental flexibility (strategies shift constantly to meet test demands). The test includes four cards of different geometric shapes and colors. Participants are then provided with 64 cards which they must classify in four categories according to the test criteria defined by the evaluator. This test analysis focuses on values of test hits, perseverations, delayed perseverations, time spent and maintenance errors [35]. The best performance requires that the subject complete all four categories, with the fewest perseverative errors.

Tower of Hanoi. This test evaluates the ability to plan and sequence actions. It consists of one base and three stakes, with three or four pieces, varying sizes, arranged on one of the side stakes. The evaluated must transfer the chips from the initial configuration on the first stake to the second. Only one chip can be transferred at a time. A chip cannot be placed on top of a smaller chip. Chips must be deposited before transferring another. This test provides scores based on the number of moves and time taken [35]. Fewer movements and less time mean better performance.

Stroop Test. Evaluates an individual’s ability to inhibit an automatic response and select an alternative one. The test involves ink words written in a different color from that in which they are read. This Stroop test is a variant of the original, and includes a version A and a version B. In version A, the subject must read the words and say the color of those that appear underlined. In version B, the subject must read the words in the first column and say the color of the words in the second column, and so on until all columns are completed. The maximum number of correct responses is 84 in both versions. This test provides hits, execution time, and Stroop errors for both versions [35]. Higher time values indicate worse performance.

All the neuropsychological test scores are normalized according to age and schooling, according to BANFE manual [35].

#### 2.3.2. Emotional State and Regulation Assessment

State-Trait Anxiety Inventory. Mexican version of the original STAI with 40 items, Likert scale, and four response categories. Designed to measure state and trait anxiety. Items are rated on a scale from ‘Not at all’ to ‘Very much’. Higher scores indicate greater anxiety. The inventory yields raw scores based on the responses, converted to T-scores. Scores are classified into three anxiety levels: low (less than 30), medium [30,31,32,33,34,35,36,37,38,39,40,41,42,43,44] and high (45+). Mexican version has shown good internal consistency, with Cronbach’s α = 0.87 [38].

State-Trait Depression Inventory. Self-assessment test containing two forms of depression (now and regularly), like the IDARE. It is a Likert-type test ranging from “Not at all” to “Very much” and consists of 42 items in total: 20 corresponding to depression as a state and 22 to depression as a trait. This instrument has a sensitivity index of over 70% and a reliability of 0.87 Cronbach’s alpha value [39].

TMMS. Spanish adaptation of the Trait Meta-Mood Scale, an instrument designed to assess perceived emotional intelligence. The scale comprises 24 items, presented on a Likert scale ranging from “strongly disagree” to “strongly agree,” divided into three subscales: attention to emotions, emotional clarity, and emotional repair. The internal consistency of the scale was found to be 0.90, as indicated by Cronbach’s alpha value [40].

### 2.4. Procedure

This study was submitted to the ethics committee of the Faculty of Sports Organization at the Autonomous University of Nuevo León, with identification number REPRIN-FOD-167. Phase 1. Reading and signing of the informed consent. Application of the Moca cognitive screening test for inclusion criteria of the participants. In the case of underage participants (*n* = 8), informed consent was obtained from the parents and informed assent from the players. Phase 2. Completion of questionnaires pertaining to their sociodemographic data including information regarding their age, position on the field, and time practicing football. Phase 3. Evaluation of psychoaffective variables: anxiety, depression, and emotional regulation were administered. Phase 4. Evaluation of cognitive performance: The Iowa Gambling Task, the Hanoi Tower, the Corsi Cube Test, the Stroop Test, and the Wisconsin Card Sorting Test were administered. The evaluations were conducted in individual sessions, each approximately 30 min in duration.

### 2.5. Statistical Analysis

The SPSS statistical package version 26 was employed to conduct descriptive and inferential analyses. The descriptive analysis was mean and standard deviation, for the evaluated psychological and neuropsychological variables. Spearman’s rank-order correlations were calculated, and 95% confidence intervals were reported for all statistically significant associations. Because multiple correlations were examined, the possibility of Type I error cannot be excluded. Therefore, significant findings should be interpreted as preliminary and hypothesis-generating, requiring replication in larger samples.

Given the relatively small sample size (*n* = 28), the present study should be considered exploratory. An a priori power analysis was not conducted because participant recruitment was constrained by the number of professional players available from the participating club during the competitive season. Therefore, findings should be interpreted cautiously and considering preliminary evidence of associations between emotional and cognitive variables.

## 3. Results

### 3.1. Participant Characteristics

The sample consisted of 28 professional female football players. Participant characteristics, including age, educational level, and years of football practice, are presented in Table 1.

### 3.2. Neuropsychological Variables

Table 2 summarizes participants’ performance across all neuropsychological measures. Overall, players demonstrated average performance in planning and working memory tasks according to the normative values provided by the respective instruments. Inhibitory control scores were generally average to above average, particularly for execution time measures in the Stroop task.

In contrast, decision-making performance assessed through the Iowa Gambling Task showed standardized scores below the normative average for both total points and percentage of risky cards selected. Similarly, the standardized number of correct responses in the Wisconsin Card Sorting Test was below average, whereas perseverative errors, maintenance errors, and execution time remained within the average or above-average ranges.

Regarding working memory, participants obtained an average standardized score in the Backward Corsi Cubes Test. The mean number of retained items and correct trials represented approximately half of the maximum achievable scores in the task.

**Figure 1:**
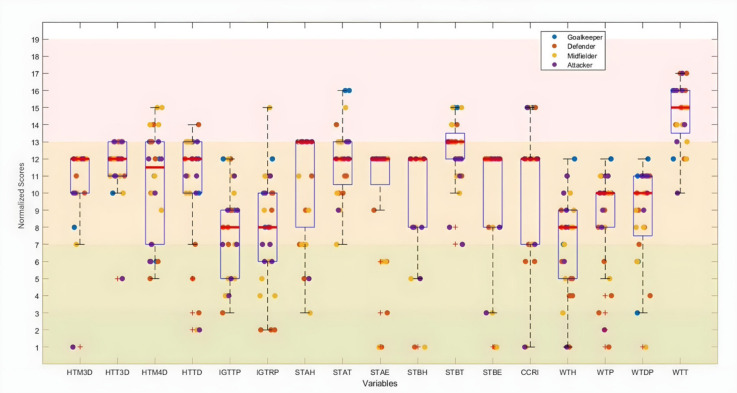
Distribution of the participants’ normalized scores in the neuropsychological tests. Note: The standardized mean score for this test is 10. Most of the scores evaluated in this study were above this mean, HTM3D = Hanoi Tower Movements 3 Disk; HTT3D = Hanoi Tower Time 3 Disk; HTM4D = Hanoi Tower Movements 4 Disk; HTTD = Hanoi Tower Time 4 Disk; IGTTP = Iowa Gambling Task Total Points; IGTRP = Iowa Gambling Task Risk Percentage; STAH = Stroop Test A Hits; STAT = Stroop Test A Time; STAE = Stroop Test A Errors; STBH = Stroop Test B Hits; STBT = Stroop Test B Time; STBE = Stroop Test B Errors; CCRI = Corsi Cubes Retained Items; WTH = Wisconsin Test Hits; WTP = Wisconsin Test Perseverations; WTDP = Wisconsin Test Delayed Perseverations; WTT = Wisconsin Test Time. Boxes represent the interquartile range (IQR), the horizontal red line within each box indicates the median, and the whiskers represent the range of non-outlying observations. The “+” symbols indicate individual observations identified as outliers by the MATLAB boxplot function. Different colors are used to distinguish the neuropsychological variables and do not represent different groups or statistical comparisons.

Descriptive statistics for all neuropsychological variables are presented in Table 2 and the distribution of neuropsychological test scores is summarized in Figure 1.

### 3.3. Emotional Variables

#### 3.3.1. Depression

**Figure 2:**
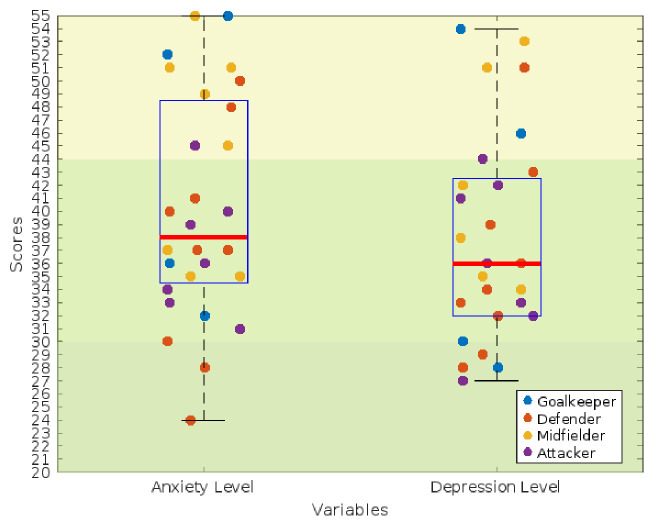
Distribution of the anxiety and depression levels reported by the IDERE and IDARE’s scores. Boxes represent the interquartile range (IQR), the horizontal red line within each box indicates the median, and the whiskers represent the range of non-outlying observations.

The IDERE-T was used to evaluate depression levels. Descriptive analyses of the 28 players’ scores yielded a mean of 38.04 (±7.87). This value indicates that, on average, participants tended to endorse response options located around the middle range of the self-report scale. Most players’ scores were concentrated in the lower-to-middle portion of the questionnaire score distribution (see Figure 2).

#### 3.3.2. Anxiety

Anxiety levels were evaluated using the Trait and State Anxiety Inventory (IDARE) in the Trait version. The descriptive analysis yielded a mean (SD) score of 40.21 (±8.54), suggesting that participants generally reported scores around the middle range of the self-report scale. Score distribution showed substantial variability across players, with most participants clustering around the central portion of the scale (see Figure 2).

#### 3.3.3. Emotional Regulation

The participants’ scores in Emotional Regulation factor exhibited a mean (SD) of 26.96 (±6.73) points. The mean (SD) score in Attention factor was 23.82 (±6.15). While the participants’ mean (SD) score in the Clarity factor was 26.57 (±6.59).

### 3.4. Correlations

**Figure 3:**
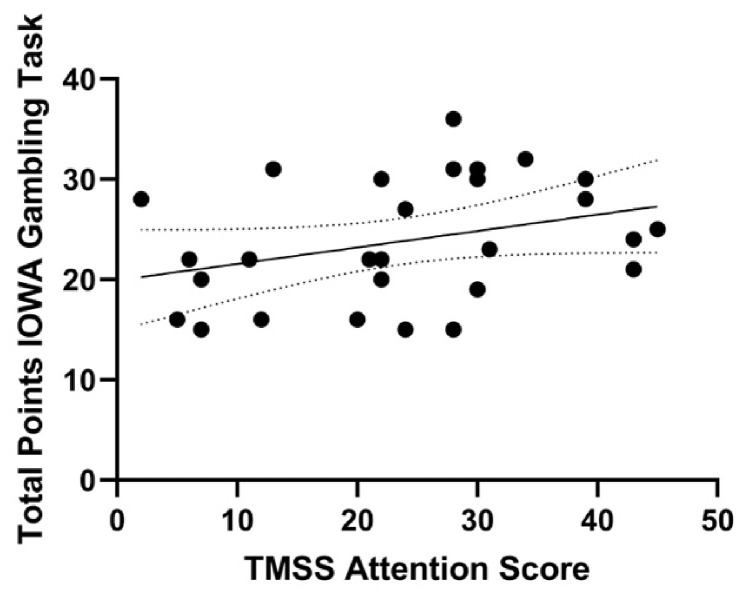
Scatter plot showing the relationship between emotional attention (TMMS-24) scores and total Iowa Gambling Task (IGT) scores in professional female football players (*n* = 28). The solid line represents the fitted regression line, and the dashed lines indicate the 95% confidence interval around the fitted regression. Each point represents one participant.

**Figure 4:**
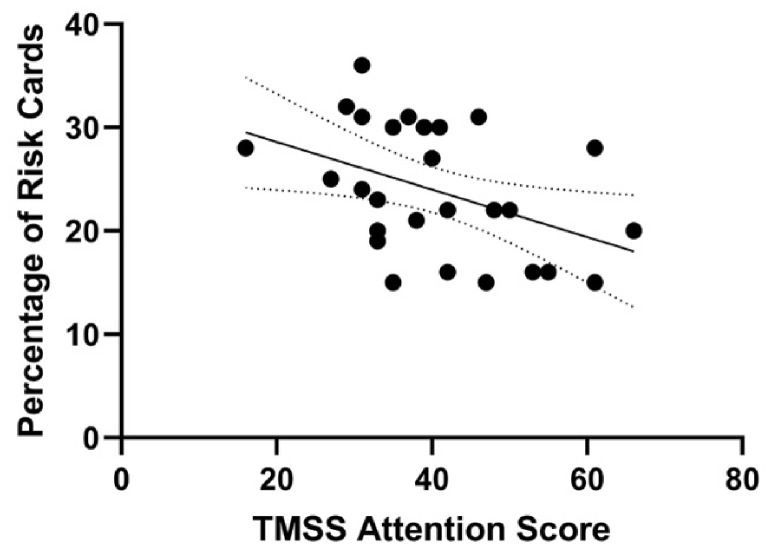
Relationship between the attention to emotions score and the percentage of risky cards on the Iowa Gambling Task. The solid line represents the fitted regression line, and the dashed lines indicate the 95% confidence interval around the fitted regression. Each point represents one participant.

To probe the hypothesis of the study the correlations between the neuropsychological and psychoaffective variables were established. As illustrated in Figure 3, the total scores obtained in the test exhibited a statistically significant positive correlation between the total points in the Iowa Gambling Task and the TMMS emotional attention score (see Table 3). Additionally, a negative correlation was identified between the percentage of risk cards and the TMMS attention score (see Table 3 and Figure 4).

**Figure 5:**
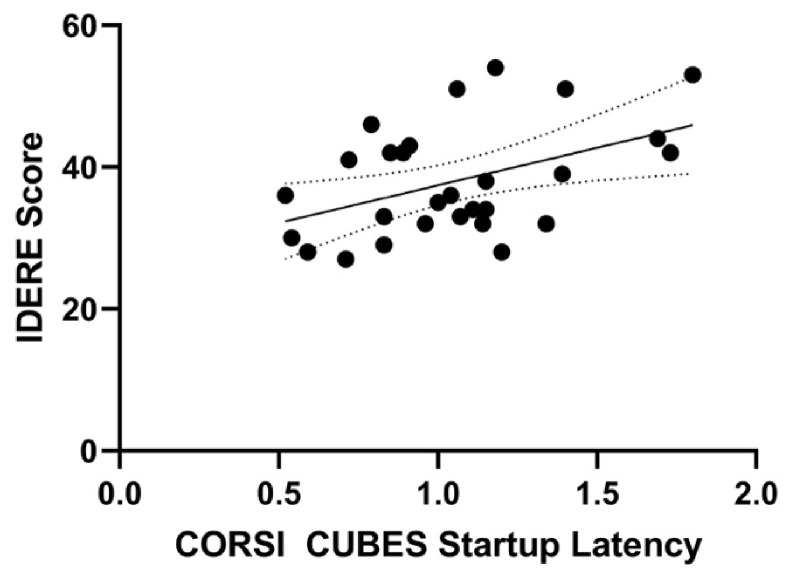
Relationship between the depression inventory scores and the initiation latency of the Corsi Cubes Test. The solid line represents the fitted regression line, and the dashed lines indicate the 95% confidence interval around the fitted regression. Each point represents one participant.

As shown in Table 3 positive correlation was found between the onset latency of the Corsi cube test (MT), and the scores obtained by the participants on the depression index, as measured by the IDERE (see Figure 5).

Furthermore, a correlation was observed between the scores obtained in the psycho-affective tests. A negative correlation was observed between the total score of anxiety as a trait and the clarity of emotions score, with a coefficient of rho = −0.41 (*p* < 0.03). While a negative correlation was observed between depression level and both emotion clarity (rho = −0.56, *p* < 0.01) and emotion regulation (rho = −0.53, *p* < 0.01). As an exploratory and control analysis, correlations were made between the scores of all the tests and the time spent practicing football of the participants, none of which was statistically significant (*p* > 0.05).

## 4. Discussion

The aim of the study was to determine if there was a relationship between emotional state, emotional regulation, and performance on tasks related to neuropsychological processes in professional female football players. Our main hypothesis is that a relationship exists between emotional states and performance on tasks related to executive functions, as well as a relationship between emotional regulation and executive function scores. We observed a positive association between emotional attention and total points obtained on the Iowa Gambling Task (rho = 0.36). However, this finding should be interpreted cautiously given the exploratory nature of the study, the limited sample size, the absence of correction for multiple comparisons, and the fact that the confidence interval included zero. Emotional attention levels also showed a moderate negative association with the percentage of risky cards selected (rho = −0.47), suggesting that greater attention to emotions may be related to more conservative choices during the task. This result supports the study’s hypothesis that emotional regulation would be related to neuropsychological processes (in this case, decision-making). 

Decision-making in sports like football involves comparing and evaluating stimuli perceived on the field [41]. Stimuli can include the ball, teammates, opponents, and open spaces. After assessing these factors, players can make a decision aligned with their objectives [42]. This aligns with the idea that tactical decisions in a play are bounded by team strategy and individual momentary objectives [43]. The relationship between these variables aligns with the findings of Shams et al. [44], who report that the valence athletes give to their emotions influences their decision-making approach. 

Emotional attention is a component of emotional regulation that has been associated with physical and cognitive performance. On the TMMS scale, the attention component reflects the awareness degree of participants regarding the emotions they experience [45]. In situations of fatigue, athletes with stronger emotional regulation skills have been reported to show better decision-making performance than those with lower emotional regulation skills [46]. The decision-making test in this study aims to maximize points while avoiding losses. Participants decide whether to take risks or not, as high-point cards also incur the highest losses. The participants took risks over 40%, indicating they do not consider consequences. This aligns with Hultman et al. [47], who described some relying on intuition, then considering advantageous options regardless of risks.

Some individuals anticipate and feel anxious about potential outcomes, choosing safer options [48]. In this study, the percentage of risk cards chosen by players was below the mean, suggesting they did not anticipate the consequences of choosing higher-denomination cards. However, these results may be influenced by the nature of activities performed by female football players. They are constantly engaged in decision-making in a match, requiring taking risks to achieve certain benefits [49].

One possible explanation of this result is that decision-making processes observed in laboratory tasks may share certain cognitive mechanisms with decision-making demands commonly encountered in football. Effective decision-making is an important point here [50]. Due to time limitations, recall procedures cannot be used and the current situation cannot be compared to past ones [51]. The conceptions of knowledge most applicable to action in a given situation tend to align more closely with procedural knowledge and emotions generated by similar previous occasions [52]. The pressure environments in which football players operate may influence their emotions and may be associated with differences in their tactical decision-making [53] and influencing their motivation for these selections [54].

Emotional consequences are a key factor in many of these decisions. Players remember what they have done well and what they have omitted because they keep the event and its results in memory through emotion, which is the effect of somatic markers [52]. The somatic marker theory proposes that emotions guide people’s decisions, including athletes. This means that when facing a new decision-making situation, athletes choose an option that brings reinforcing or aversive emotional consequences. As a result, this “somatic marker” is retrieved when confronted with similar conditions, making the decision-making process more efficient [25].

Another finding of the present study was a positive association between depression scores and onset latency in the Corsi Block Test, a measure related to working memory (rho = 0.38; *p* = 0.04). Higher depression scores were associated with longer initiation times before responding to each trial. However, given the exploratory nature of the study and the limited sample size, this association should be interpreted with caution until replicated in larger samples. The onset latency measure may reflect the efficiency with which participants initiate the working-memory process required to remember the sequence of cubes and reproduce it in reverse order [55].

The findings support the hypothesis that depression is linked to neuropsychological variables, such as working memory. The results are consistent with other research, including Andreotti et al. [56] outcomes, who also found an association between depression and working memory. Good working memory is often linked to lower levels of depression in athletes [57]. Working memory aids in on-field positioning and analyzing plays [58], motor learning [59], and talent scouting in football [60].

Working memory in football is crucial due to the constant change in tactics and visual stimuli. Therefore, it is expected that female football players can make quick decisions with the help of this cognitive skill [61]. It has been found that stimulated and properly functioning working memory is associated with lower levels of depression [62]. Chen et al. [63] state that working memory impairments in individuals with depression reinforce the cyclical relationship between the two, as working memory processing may be related to cognitive biases due to its involvement in rumination. Those with high levels of depression may experience difficulties in open-ended situations and continuous performance scenarios (such as football) that require working memory [64].

Planning and cognitive flexibility were the processes that did not have a relationship with emotions. Trait anxiety levels appeared to have no relationship with cognitive performance in any of the evaluated processes. This finding contrasts with results from other studies that have described anxiety as an influencing factor in the performance of neuropsychological tasks [65].

In general terms, the results obtained in this study are consistent with those of Castillo-Rodríguez et al. [24], who stated that emotional states and their regulation are associated with cognitive performance in processes such as decision-making in athletes. This result matches the proposal by Zelazo & Muller [9], who categorize decision-making as a hot executive function because it requires emotional involvement to evaluate immediate or long-term rewards. 

Hot EFs are often involved in emotional regulation and risky decision-making tasks. However, they can also interact with cold executive functions depending on contextual information and individuals’ motivations [66]. Research describes that cold executive functions can become hot when there are elevated levels of depression [67], reporting that working memory is often compromised in such cases [68], as presented in this study.

The scores on the neuropsychological tests fell within or above the standardized mean, except for certain factors related to decision-making and cognitive flexibility. These results are partially similar to those obtained by Vaughan and Laborde [53], who found working memory scores above the mean, as well as those reported by Verburgh et al. [69], who identified both elite and amateur players to achieve scores at or above the standardized mean.

The scores above the mean in inhibitory control obtained by the participants align with the findings reported by Wang et al. [70], who states that athletes develop their inhibitory control which has been associated with athletic success. The scores obtained by the participants may be influenced by the quality of training that the football players received during their development before transitioning to the professional level, as well as by the type of training and strategies [61].

The predominance of anxiety scores clustered around the middle portion of the self-report scale is consistent with the findings of Kristjansdottir et al. [33], who saw moderated and high levels of anxiety in professional female football players. This may be explained by the context in which the players live, the demands of being a professional player in a growing league and the constant struggle for salaries that are sufficient for their needs. The concentration of anxiety scores in the middle-to-upper range of the self-report scale of this study aligns with the findings of Ramírez-Goerke et al. [71]. In contrast, Sobrinho et al.’s study [72] found that young football players scored lower levels of depression. 

Emotional regulation measured by the TMMS scale yields scores for the factors of emotional attention, emotional clarity, and emotional regulation. The results show higher scores in regulation compared to the other two factors; these findings are similar to those reported by Chirinos-Lizárraga [73], who found that the regulation factor has the highest scores in emotion regulation. Emotion regulation in the TMMS-24 is associated with individuals’ belief in their ability to regulate negative emotional states and prolong positive ones [40]. Therefore, this is a predominant skill among the participants in this study, as it not only received the highest score, but also fell within an appropriate range.

In football, players must react quickly to teammates’ and opponents’ actions, inhibit behaviors such as passing, dribbling and shooting, and adapt to the game’s situations. It is essential to understand how these variables interact. Studying the relationship between emotions and cognitive processes may contribute to a better understanding of factors associated with cognitive functioning in athletes [74].

Studies demonstrate that emotional states affect cognitive performance [65] while sports performance is often negatively influenced when athletes feel anxious or depressed [75]. Therefore, it is important to understand the relationship between emotions, their regulation and cognitive functions, as players’ psychological state can modify their motivational and attentional parameters depending on the scenario [52]. In addition to relying on cognitive skills, athletes can draw on emotional intelligence to regulate their emotions and behavior [76].

This study contributes to the growing body of knowledge on the intersection of emotional regulation, mental health, and cognitive performance in athletes. Nevertheless, because the present study did not include amateur athletes or non-athlete controls, the findings should be interpreted as evidence of associations within professional female football players rather than evidence that these relationships are unique to this population. Its findings highlight potential associations between emotional intelligence, emotional regulation, and cognitive performance, which is relevant in terms of mental health issues in athletes. Future studies may determine whether these associations have practical implications for athlete support programs.

This study did not include any non-athlete control group to contrast emotional and neuropsychological variables reported by the participants; therefore, it is not possible to determine whether the observed relationships between emotional variables and executive functions are specific to professional female football players or reflect broader patterns found in the general population. Only reports of psychiatric history and traumatic injury given by the players were considered as part of the exclusion criteria. There were no evaluations for it.

Additional factors that may influence both emotional states and neuropsychological performance were not systematically assessed, including sleep quality, fatigue, training load, time since the last match, medication use, current stress levels, and concussion history. Although age and educational level were considered through the normalization procedures of the neuropsychological tests, and psychiatric history and traumatic injuries were addressed through exclusion criteria, future studies should incorporate these variables directly to better understand their potential influence on the observed associations.

The menstrual cycle phase and hormonal status of the players were not assessed and therefore could not be controlled in the analyses. Although hormonal fluctuations have been proposed as a potential source of variability in emotional states and cognitive performance, current evidence regarding their effects on cognition remains mixed and inconclusive. Recent reviews have reported small, inconsistent, and often non-replicable effects across cognitive domains, suggesting that menstrual cycle-related differences may be less robust than traditionally assumed. Nevertheless, menstrual cycle phase could be considered in future studies as an exploratory variable, particularly in relation to the perceived emotional states.

The relatively small sample size may have reduced statistical power and increased the instability of correlation estimates. Consequently, the observed associations should be interpreted as exploratory rather than definitive. Future studies including larger samples from multiple clubs and leagues are needed to determine the robustness and generalizability of these findings.

Future studies should consider documenting menstrual cycle phase and hormonal contraceptive use to further examine their potential influence in female athletes. An important methodological consideration is that trait measures of anxiety and depression were used in the present study. These instruments assess relatively stable emotional dispositions rather than transient emotional states that may vary according to contextual factors such as competition demands, fatigue, recent performance, or situational stress. Consequently, the observed associations should be interpreted as reflecting relationships between enduring emotional tendencies and cognitive-task performance. Future studies should incorporate both trait and state measures to determine whether situational emotional fluctuations are differentially associated with executive function performance in female football players.

The lack of a standardized procedure for neuropsychological football player evaluations has led to inconsistent findings. Standardizing these evaluations is recommended for future studies. A larger sample size and comparison of sexes at different competitive levels may help spot potential performance differences between these groups. Additionally, future studies should be required to determine how these variables change throughout their career. These functions help with decision-making when immediate gratification and long-term rewards are in conflict [9].

## 5. Conclusions

This study explored associations between emotional variables and cognitive-task performance in professional female football players. Greater emotional attention was associated with decision-making outcomes on the Iowa Gambling Task, whereas higher depression scores were associated with longer onset latencies in a working-memory task. Although these findings do not establish causal relationships, they suggest that emotional and cognitive processes may be interconnected in this population. These findings suggest associations between emotional processes and cognitive functioning in professional female football players and highlight the potential relevance of emotional factors in football performance contexts. Future research should further examine the role of emotional and cognitive variables in football-specific performance outcomes.

## Figures and Tables

**Table 1:** Descriptive characteristics of the participants.

	Age	Education	Years of Football Practice
Mean	22.2	13.6	13.5
SD	4.75	3.13	5.59
Minimum	16	9	1
Maximum	30	19	24

SD = Standard Deviation.

**Table 2:** Neuropsychological performance of professional female football players.

Executive Function	Test	Outcome Variable	Raw Score Mean ± SD	Standardized Score Mean ± SD	Interpretation
Planning	Tower of Hanoi (3 disks)	Number of movements	9.18 ± 4.43	10.89 ± 2.34	Average
Planning	Tower of Hanoi (3 disks)	Execution time (s)	24.11 ± 19.10	11.67 ± 1.58	Average
Planning	Tower of Hanoi (4 disks)	Number of movements	25.18 ± 8.87	10.60 ± 3.19	Average
Planning	Tower of Hanoi (4 disks)	Execution time (s)	83.79 ± 63.94	11.46 ± 3.44	Average
Decision Making	Iowa Gambling Task	Total points	23.79 ± 12.37	7.71 ± 2.69	Below average
Decision Making	Iowa Gambling Task	Risk card percentage	40.79 ± 11.41	7.89 ± 3.25	Below average
Inhibitory Control	Stroop A	Hits	82.89 ± 1.59	12.76 ± 3.19	Above average
Inhibitory Control	Stroop A	Execution time (s)	69.46 ± 12.88	12.07 ± 2.07	Above average
Inhibitory Control	Stroop A	Errors	0.64 ± 1.25	11.78 ± 3.86	Average
Inhibitory Control	Stroop B	Hits	83.32 ± 1.46	11.34 ± 3.37	Average
Inhibitory Control	Stroop B	Execution time (s)	59.86 ± 10.16	13.00 ± 3.63	Above average
Inhibitory Control	Stroop B	Errors	0.64 ± 1.47	12.42 ± 1.91	Above average
Working Memory	Backward Corsi	Maximum span	5.50 ± 1.55	11.35 ± 4.04	Average
Working Memory	Backward Corsi	Correct trials	7.36 ± 2.45	—	—
Working Memory	Backward Corsi	Onset latency (s)	1.05 ± 0.33	—	—
Cognitive Flexibility	Wisconsin Card Sorting Test	Hits	38.86 ± 9.12	6.96 ± 2.87	Below average
Cognitive Flexibility	Wisconsin Card Sorting Test	Perseverations	6.75 ± 4.51	9.53 ± 2.93	Average
Cognitive Flexibility	Wisconsin Card Sorting Test	Maintenance errors	1.04 ± 1.17	9.85 ± 2.81	Average
Cognitive Flexibility	Wisconsin Card Sorting Test	Execution time (s)	196.11 ± 61.40	14.67 ± 1.81	Above average

Note. Standardized scores are based on the normative values provided by the respective neuropsychological instruments. Scores between 8 and 12 were considered average, scores below 8 below average, and scores above 12 above average.

**Table 3:** Significant correlations between emotional variables and neuropsychological performance.

Emotional Variable	Neuropsychological Variable	Rho	95% CI	*p*
Emotional Attention	IGT Total Points	0.36	[−0.01, 0.65]	0.05
Emotional Attention	Risk Cards %	−0.47	[−0.72, −0.12]	0.01
Depression	Corsi Latency	0.38	[0.01, 0.66]	0.04

rho = Spearman’s rank correlation coefficient; CI = confidence interval. Results should be interpreted as exploratory due to the limited sample size and the number of correlations examined.

## Data Availability

The raw data supporting the conclusions of this article will be made available by the authors on request.
